# Social network-based group intervention to promote HIV prevention in Uganda: study protocol for a cluster randomized controlled trial of *Game Changers*

**DOI:** 10.1186/s13063-022-06186-z

**Published:** 2022-03-28

**Authors:** Glenn J. Wagner, Laura M. Bogart, Harold D. Green, Erik D. Storholm, David J. Klein, Ryan K. McBain, Richard Serunkuuma, Kuraish Mubiru, Joseph K. B. Matovu, Stephen Okoboi

**Affiliations:** 1 RAND Corporation, 1776 Main Street, P.O. Box 2138, Santa Monica, CA 90407-2138 USA; 2 Indiana University School of Public Health, Bloomington, IN USA; 3 San Diego State University, San Diego, CA USA; 4 Infectious Diseases Institute, College of Health Sciences, National Forum of People Living with HIV/AIDS Networks in Uganda, Kampala, Uganda; 5 School of Public Health, Makerere University, Kampala, Uganda; 6 Busitema University Faculty of Health Sciences, Mbale, Uganda; 7 Infectious Diseases Institute, College of Health Sciences, Kampala, Uganda

**Keywords:** HIV, Prevention advocacy, Group intervention, Cluster randomized controlled trial, Uganda

## Abstract

**Introduction:**

Innovative strategies are needed to disseminate HIV prevention messages across communities efficiently, as well as reduce HIV stigma while promoting HIV prevention. This randomized controlled trial will evaluate the efficacy of a social network-based group intervention, *Game Changers*, which trains persons living with HIV (PLWH) to encourage members of their social network to use HIV protective behaviors

**Methods:**

PLWH in HIV care for at least 1 year will be randomly assigned to receive the 8-session group advocacy training intervention or no-intervention control group. Each enrolled PLWH (index participant) will be asked to recruit up to four social network members (alter participant). Assessments will be administered at baseline and months 6, 12, and 18 to both index and alter participants. The primary outcomes are HIV testing and condom use among alter participants; secondary outcomes are engagement in HIV prevention advocacy and internalized HIV stigma among index participants. Repeated-measures multivariable regression analyses will be conducted to compare outcomes between the intervention and control arms, in addition to a cost-effectiveness evaluation.

**Discussion:**

This social network-based approach to HIV prevention is particularly timely in the era of biomedical interventions, which require widespread penetration of effective HIV prevention and care messaging into communities. Positioning PLWH as central to the solution for controlling (vs. causing) the HIV epidemic has the potential to reduce HIV stigma and improve prevention outcomes at the individual and network levels.

**Trial registration:**

ClinicalTrials.gov NIH Clinical Trial Registry NCT05098015. Registered on October 18, 2021.

## Introduction

In Uganda, HIV prevalence is estimated to be 5.5% among those aged 15–64 [1–4]. Despite some progress against the epidemic, political and cultural barriers, including limited government funding and HIV stigma [5], impede HIV prevention and have led to a stagnate HIV prevalence [3]. Innovative solutions are needed to disseminate HIV prevention messages and information across communities efficiently, as well as reduce HIV stigma while promoting HIV prevention.

One promising approach is to empower persons living with HIV (PLWH), who are successfully managing their HIV disease, to act as change agents by encouraging members of their social network to use HIV protective behaviors. Research suggests that as PLWH receive ART and stabilize their health, they are motivated to protect people in their social network and engage in prevention advocacy (i.e., encourage friends and family to seek HIV testing and care, and reduce HIV risk); however, the quality of this advocacy is hampered by challenges related to message content, style and timing of delivery, and the selection of appropriate recipients for advocacy [6, 7]. With effective advocacy training, mobilizing PLWH to be change agents within their networks has the potential to be a “game changer” for HIV prevention, particularly in high-prevalence settings such as Uganda where virtually every family is touched by HIV. Ugandan PLWH have dense, interconnected networks [8], and PLWH have disclosed to many people in their network [9], suggesting that transfer of HIV prevention messages and knowledge can be safe and efficient. Further, PLWH have access to at-risk individuals within their networks and can be influential and credible in conveying prevention messages to family and friends, given their close relations and their ability to exemplify the benefits of HIV testing and care on health [10].

**Fig. 1:**
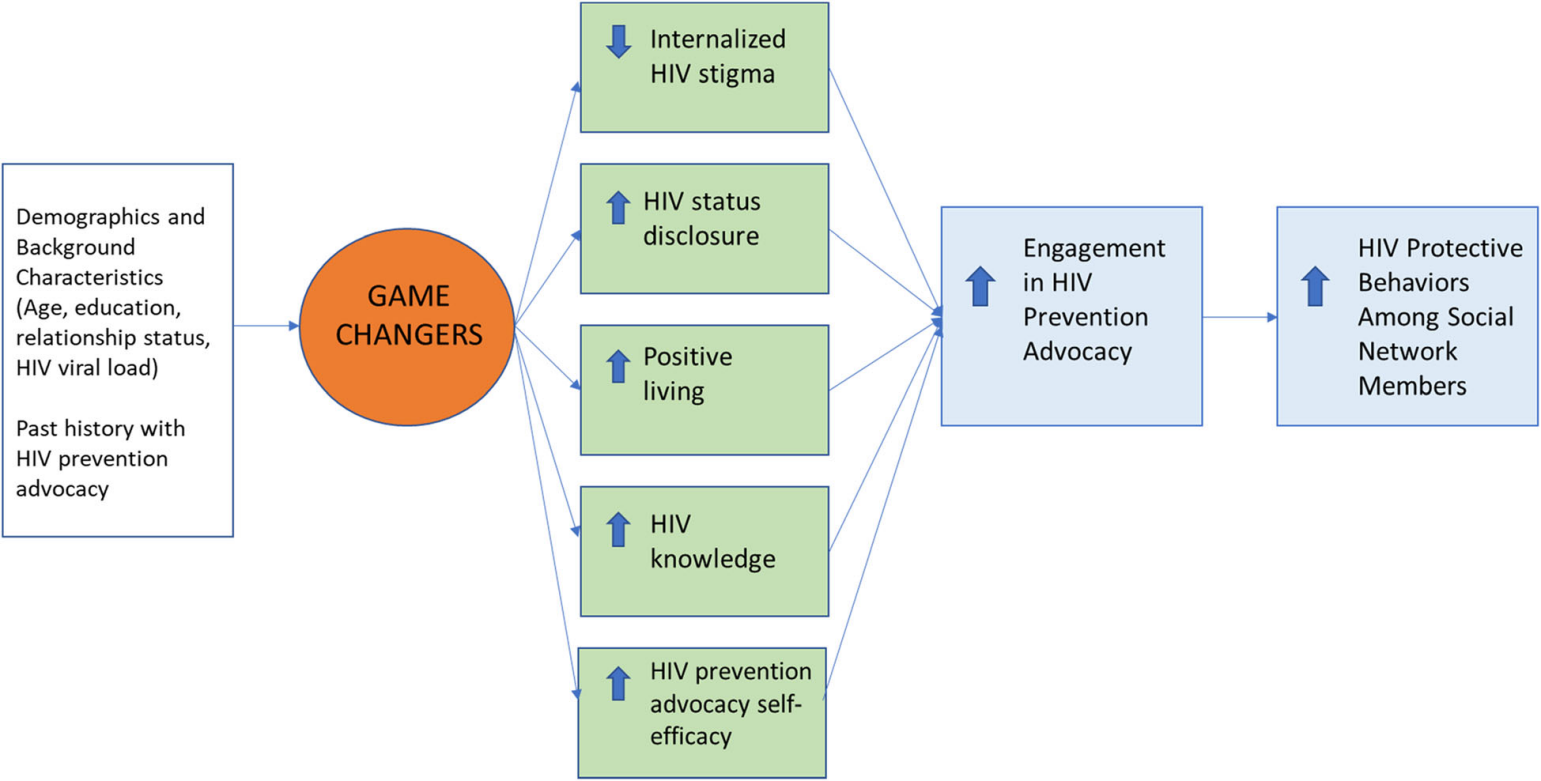
Conceptual framework for promotion of HIV prevention advocacy among persons living with HIV to affect HIV protective behaviors among social network members

We recently developed a network-based advocacy group intervention, *Game Changers*, that mobilizes PLWH to act as change agents for HIV prevention within their social network, through mechanisms that aim to reduce internalized HIV stigma, increase HIV disclosure skills, healthy living (e.g., through HIV treatment adherence), knowledge of HIV fact and misconceptions, and advocacy skills (see Fig. 1) [11]. A randomized controlled pilot in Uganda, conducted over 1 year starting in 2018 with 99 PLWH and 58 of their social network members, showed that the intervention resulted in reduced internalized HIV stigma and increased HIV disclosure and engagement in advocacy among intervention recipients, and increased HIV testing and condom use among their network members [12].

To perform a larger, more rigorous evaluation of *Game Changers*, we will conduct a randomized controlled trial of this 8-session peer-led group intervention. Our primary hypothesis is that the social network members of recipients of *Game Changers* will report greater HIV testing and more condom use, compared to network members of control participants. Our secondary hypothesis is that PLWH in the intervention arm will report less internalized HIV stigma, and engagement in HIV prevention advocacy, compared to PLWH in the control group. We will also conduct a cost-effectiveness analysis of the intervention.

## Methods

### Study design

This study is an individually randomized group-treatment superiority trial using a clustered design [13]. We have used the SPIRIT reporting guidelines to document the study methods in the study protocol [14]. We will recruit 210 PLWH and randomize 105 to receive the intervention and 105 to the non-intervention control group. A non-intervention control was used, as opposed to an attention control that would focus on the intervention participants, as this attention would not be comparable to the control for attention needed for effects on social network members (who provide data for the primary hypothesized outcomes). Participants in both groups receive care as usual from the study site, which does not involve any training related to advocacy. A blocked 1:1 randomization design with stratification by gender (with randomly alternating blocks of 2, 4, and 6 to prevent anticipation of condition) will ensure balance across arms. The statistician used a random number generator to devise a randomization log and a set of sealed numbered randomization envelopes which is used by the study coordinator to enroll participants and assign the intervention or control. The coordinator and data collectors are blind to the assignment until after the baseline assessments are completed; after the random assignment is revealed, the only blind party is the data analyst. Each PLWH is asked to recruit up to four social network members (also referred to as “alters”) to whom they have disclosed their HIV status; we expect to enroll approximately 736 alters in total (368 per arm). All participants will complete assessments at baseline and 6, 12 and 18 months post-baseline. Participants receive 30,000 Ugandan Shillings (about $8 USD) per assessment and intervention session attended, to cover transportation costs. All study participants will provide written informed consent.

### Trial coordination

The day-to-day study activities are coordinated directly by the project director, in tandem with the study coordinator, and all activities are overseen by the senior investigators. The study coordinator and data collectors recruit and track participants, as well as administer data collection tools. The study coordinator also coordinates the logistics of planning the intervention sessions. The project director supervises and provides direct oversight of the study coordinator and data collectors, whom she meets with weekly; she also supervises the intervention facilitators, and meets with them weekly in a separate meeting. The senior investigators provided oversight of the project director and study coordinator via twice-a-month meetings during which progress with study activities are reviewed and any challenges are addressed as a team.

### Participants

All study activities and recruitment will be conducted in Kampala at The Infectious Diseases Institute (IDI), which provides outpatient HIV care to ~8000 PLWH. Eligibility criteria for PLWH include (1) age ≥ 18 years, (2) HIV-positive, (3) in HIV care for > 1 year (because they are more likely to be medically stable, adjusted to their HIV diagnosis, and have disclosed to several people, and thus more likely to be ready to engage in advocacy), (4) did not participate in the pilot study of the intervention, (5) speaks fluent Luganda, (6) health status sufficiently stable (based on medical chart review) to complete the 18-month study, (7) no signs of significant cognitive impairment (based on interviewer observation), and (8) a partner/spouse or household member living with HIV is not already enrolled in the study as an index (PLWH) participant. Eligibility criteria for alters include (1) age ≥ 18 years, (2) referred by a PLWH enrolled in the study, (3) knows the referring PLWH’s HIV serostatus, (4) speaks fluent English or Luganda, (5) health status is sufficiently stable (based on interviewer observation) to complete the 18-month study, and (6) no signs of significant cognitive impairment (based on interviewer observation).

### Recruitment

To recruit PLWH, the study coordinator will give a brief talk in the IDI waiting room at the start of each recruitment day, describing the nature of the study and participation involvement. Interested clients will be asked to reveal themselves to the coordinator or data collectors, who will then conduct a formal screening of their eligibility, followed by the informed consent process (including obtaining written informed consent), administration of the baseline interview, and randomization. The consent form includes a provision that indicates a version of the study data in which all identifying information is removed may be made available for use by other scientists for the purpose of further research. Given the nature of the intervention, there is no anticipated harm nor compensation for trial participation. Informed consent materials are available, on request, from the corresponding author.

**Fig. 2:**
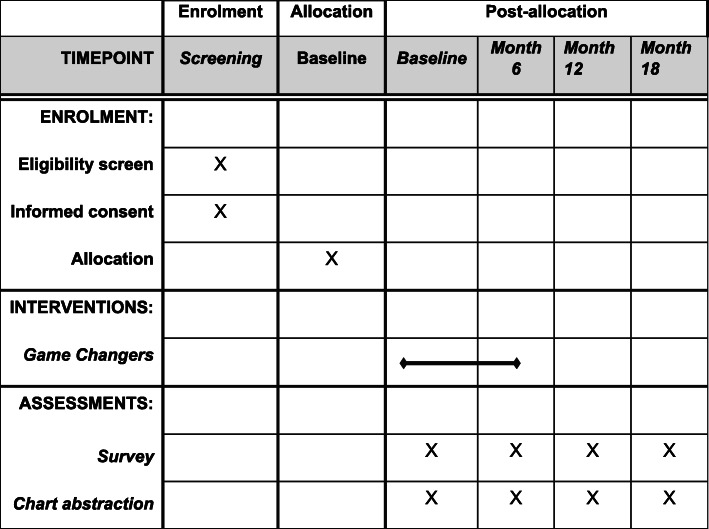
Schedule of enrolment, interventions, and assessments

To recruit alters, during the baseline interview of the PLWH index participant, we will elicit the names of 20 alters in their social network and ask the participant to identify the alters who know the index participant’s HIV serostatus. At the end of the interview, the index participant will be asked to select, from the list of alters who know their serostatus, up to seven alters whom they would be willing to refer to the study. If they refer more than four alters, we will randomly select four to target for recruitment (to limit selection bias), although we aim to recruit an average of 3.5 alters per index participant, given that we do not expect to be able to recruit 4 alters from all participants. The decision to recruit 4 is based on a need to balance interviewing enough alters to be sufficiently representative of the person’s network, while also being feasible to collect the data from the large sample of overall alters in a longitudinal design. We will request that the index participant call each selected alter at the end of the interview, and to describe the study opportunity in the presence of the coordinator, who can then immediately schedule a study visit for the alter (Fig. 2). If any of the four alters refuse to participate or cannot be reached, we will randomly select additional alters on the list of those referred from the index participant until four have agreed and enrolled; if only four were referred, we will ask the index participant to refer additional alters, if possible.

PLWH index participants will be divided into seven cohorts of 30 (within each cohort, 15 intervention and 15 control), enrolled over 12 months. We will enroll two intervention-control cohorts at a time (except for the final seventh cohort), resulting in 60 PLWH index participants (and 210 alters) enrolled (30 index and 105 alters per cohort) per wave of recruitment; each wave of recruitment will take place over 2 months, followed by a 2-month period when the intervention is implemented with the two cohorts, and no further recruitment until the next wave of recruitment (approximately 4 months after the start of recruitment in the prior wave).

To promote study retention, we collect tracking information (phone numbers, mapped addresses, contacts for family/friends with whom they have frequent contact).

### Community engagement

We will partner with two community stakeholder groups, IDI’s community advisory board (CAB) and the National Forum of People Living with HIV/AIDS Networks in Uganda (NAFOPHANU), to obtain input on the assessment instruments, intervention implementation, interpretation of results, and next steps for intervention dissemination, if it is shown to be cost-effective. IDI’s CAB and NAFOPHANU have approximately 20 members each from diverse community groups (e.g., clergy, activists, youth leaders, expert patients). Notably, the intervention facilitators are members of NAFOPHANU. Members of both groups will receive transport costs and refreshments at each meeting, which we expect to be held approximately twice a year.

### Game Changers intervention

Game Changers draws on social diffusion theory [15–18] and principles of social influence [19] to posit that behavior change can be initiated by a few, and diffused to others, through social norm change, by harnessing the power of people in the network (i.e., the PLWH recipients of the intervention) whom alters see as supportive and credible [20–24], and by engaging in HIV prevention advocacy with alters across networks [25]. The conceptual framework and hypothesized mechanisms by which the intervention is expected to have its effects are depicted in Fig. 1. To instill participants with the skills and confidence to engage in effective HIV prevention advocacy, Game Changers uses adapted HIV prevention advocacy strategies from interventions such as Mpowerment [26], and Popular Opinion Leader [24] interventions, as well as an effective parent-adolescent communication intervention developed in South Africa [24, 27, 28].

The Facilitator Manual, available in English and Luganda, was iteratively revised in the pilot following formative research (focus groups with participants and intervention facilitators) and feedback from the IDI CAB and NAFOPHANU [12]. The intervention consists of 8 weekly sessions (outlined in Table 1), with each sessions lasting about 2 h. Each session includes refreshments and is facilitated by two trained HIV-positive peer facilitators (not clinic staff or providers) with experience leading groups and HIV education. The group format facilitates an interactive process, with social reinforcement, modeling, and role plays. All sessions use sharing of experiences to build support and solidarity; group problem solving and role plays to build skills and self-efficacy; personal goal-setting for positive living, disclosure, and advocacy; and take-home activities to reinforce practice of new skills and generate personal experiences for discussion in the sessions. Role plays enable new skills to be practiced and observed and for facilitators to assess whether skills have been mastered or whether more practice is needed. Low educational levels are common, so the take-home activities do not require writing; rather, participants are asked to engage in an activity (e.g., disclosure, prevention advocacy) and report back. All groups will be mixed sex (with approximately equal numbers of men and women per group), and a male and a female facilitator will conduct each group. There are no specific criteria for discontinuing or modifying allocated intervention condition for individual participants.

**Table 1:** *Game Changers* session content

Topic	Content	Take-home activity
**1: Introduction and stigma reduction**	Introduce goals; set rules for confidentiality Introduce and define self-stigma, prevention advocacy, and disclosure decision-making Use discussion of stigma experiences and strategies for coping with stigma to model adaptive coping and promote self-compassion	*Practicing self-compassion*: Focus on a difficult experience; acknowledge and accept one’s own suffering; offer oneself self-compassion
**2: Empathy, self-compassion, and HIV disclosure**	Define empathy and self-compassion, with the aid of role plays Discuss healthy disclosure decision making; use sharing of experiences to highlight potential risks and benefits of disclosure Convey importance of establishing a basis of empathy and self-compassion, and comfort with disclosure and discussing HIV, prior to conducting prevention advocacy	*Set personal goals for disclosure:* Assess pros and cons of disclosure to at least one social network member, and practice initiating disclosure conversations
**3: Positive living, HIV facts and myths**	Share experiences with disclosure and coping with stigma since last session; provide reinforcement and problem solving of challenges Present accurate HIV information and address common HIV myths and misconceptions Discuss how credible advocacy for HIV prevention requires being able to model behaviors in one’s own life (positive living) Set personal goals related to positive living (e.g., adherence)	*Listing social network members*: In preparation for the next session, participants are asked to think about whom they consider the 20 most important people to them
**4. Introduction to social networks**	Introduce the concept of social networks as key to doing advocacy Show participants how to map their own social network and identify alters to whom they have disclosed and discussed prevention and to whom they would like to do so Define the concept of strategically positioned alters and discuss where strategically positioned alters are in participants’ network maps Use network maps and strategically positioned alters to highlight how participants can play a key role in their community through advocacy	*Set personal goals for prevention advocacy*
**5–7: Prevention advocacy skills building**	Share experiences with disclosure and advocacy since last session; provide reinforcement and problem solve challenges Discuss how advocacy protects others, and how PLWH are credible prevention messengers; validate fears and anxiety re: advocacy Discuss strategies for effective advocacy (teaching moments, open-ended questions, rephrasing); use role plays to build skills Discuss how to support alters before and after they have been tested for HIV Use role playing to practice and model effective advocacy	*Practice prevention advocacy*: Use network map to select alters to target for disclosure and advocacy, including strategically positioned alters
**8: Wrap up and review**	Share experiences with disclosure and advocacy since last session; provide reinforcement and problem solve challenges Use role plays to practice and model challenging scenarios Share experiences with program; Affirm commitment toward goals	N/A

All sessions (except session 1) begin with a review of the prior session and experiences with the take-home activity, and all sessions (except session 8) end with a review of that day’s session, take-home activity for the coming week, and an uplifting song/blessing to build solidarity

#### Facilitator training, supervision, and fidelity monitoring

Four bilingual (Luganda, English) HIV-positive peer facilitators (two men, two women) will be trained to implement the intervention. Lay peers were selected to facilitate the sessions, rather than clinic staff or providers, because of the success of other peer-led group interventions [29, 30], and the desire for the intervention to be sustainable and transportable with the need for limited resources. The training will be conducted with the full study team to facilitate mock implementation of exercises. The training will cover manual review (objectives for each session, step-by-step scripts, and key points) and group facilitation skills (building rapport, active listening, managing dominating and shy participants, and dealing with group conflict). The lead supervisor of the facilitators will observe the implementation of each session by each set of facilitators to provide feedback and further training as needed during weekly supervision. To monitor fidelity, sessions will be rated by the supervisor to measure whether objectives were met, exercises completed, level of participant engagement, difficulties encountered, and areas to improve.

### Measures

The survey assessment includes individual-level questions for both PLWH and alter participants and a social network assessment for PLWH only. Assessments will be administered on laptops using Network Canvas, a social network and survey data collection and analysis software. The assessments include the following sections, which we have refined across multiple studies in the United States and Uganda (and translated into Luganda) [12, 31, 32].

### Primary outcomes (among alter participants)

#### HIV testing

At every timepoint, alters will report whether they have been tested for HIV in the past 6 months and, if so, the date of their most recent test and result and whether they intend to get tested in the next 6 months (1, not at all, to 5, very much) or start HIV care and ART (if warranted).

#### Sexual behavior (condomless intercourse)

Sexual behavior in the past 6 months will be assessed with standard items on number of partners; number of times engaged in intercourse (vaginal or anal) and, of those, number of times a condom was used; and perceived HIV serostatus of partner(s).

### Secondary outcomes (among PLWH index participants)

#### HIV prevention advocacy

HIV prevention advocacy will be assessed with a scale we developed, measuring the extent to which participants discussed six different HIV-related topics (e.g., HIV testing, pre-exposure prophylaxis (PrEP), condom use, ART use) with people they know in the past 6 months (1 = not at all to 5 = very much).

#### Internalized HIV stigma

Internalized HIV stigma will be assessed with the 8-item Internalized AIDS-Related Stigma Scale [33].

#### Social network assessment (PLWH only)

We are using a longitudinal personal, egocentric network approach to ask participants to list first names and last initials of 20 individuals with whom they have been in communication in the past 6 months (e.g., in person, phone, text), starting with those most important to them. For each individual named, respondents provided information about demographics, HIV status, relation to and interaction frequency with the respondent, knowledge of respondent’s HIV status, and perception of use of HIV protective behaviors (condom use, HIV testing, PrEP use, ART use).

### Statistical analysis

To assess the primary and secondary hypotheses regarding intervention effects on alter and index measures, we will use a standard intent-to-treat approach, applying generalized mixed models (linear for continuous outcomes; logistic for binary outcomes) to the repeated-measures data to examine how intervention effects change over time, using indicators for study arm and each follow-up timepoint (vs. baseline), and timepoint by arm interactions to indicate whether change differs between arms. Our statistical methods will be consistent with advances in the analysis of personal network research [15, 34–36], accounting for any correlation among participants in the same intervention group sessions, and among alters referred by the same PLWH, by adjusting standard errors for statistical inference tests with a sandwich estimator as implemented in SAS v 9.4 [13, 37–40]. We will use imputation for item nonresponse and account for non-random dropouts using logistic regressions that assign weights to retained participants that are inversely proportionate to the predicted probability of the participant being retained; if dropout is random, analyses will incorporate design effects.

### Statistical power

We calculated power for alter reports of condomless sex at month 18. Based on the pilot [12], we assume 65% of control alters will report condomless sex at month 18. Starting with 736 alters and assuming ~15% attrition by month 18 (which is conservative, based on 98% alter retention over 8 months in the pilot, and < 10% attrition over 12 months in our other studies in Uganda), we will have 625 alters; accounting for within-index clustering gives us an effective sample size of 612 for ICC = .01 or 568 for ICC = .05. For ICC = .01, we will have .80 power to detect a group difference of 11.1% (i.e., an intervention rate of 53.9%), and for ICC = .05, we will have power to detect a 11.5% group difference. These differences represent small effect sizes (Cohen’s *d* =.23). For PLWH index participants, using pilot data, we estimate the ICCs of our secondary outcomes to be small-to-moderate (0–.16), and assume 15% attribution and 7 intervention groups (either Game Changers or control), with an average cluster size of 12.9 (90 intervention participants/7 groups) [13, 39, 40]. After accounting for within-group correlations, we will have 80% power to detect medium differences (.41–.58 standard deviation, based on the outcome) between the study arms at follow-up.

#### Cost-effectiveness analysis (CEA)

We will compare Game Changers to a no-intervention control group in terms of the marginal cost of decreasing alters’ condomless intercourse [41]. According to standard convention [41], we will define the incremental cost-effectiveness ratio as the difference in per-capita cost of the intervention versus control group divided by the difference in their average effectiveness:

Eq. 1 $$ ICER=\frac{\mu_{C2}-{\mu}_{C1}\ }{\delta_{e2}-{\delta}_{e1}} $$

where μ_c2_ is the per-capita cost of *Game Changers*, μ_c1_ is the per-capita cost of the control group, δ_e2_ is the percentage of alters reporting any condomless intercourse among alters affiliated with the intervention group, and δ_e1_ is the percentage of alters reporting any condomless intercourse among alters affiliated with the control group. We will estimate confidence intervals using bootstrap methods [42].

CEA will be performed from the provider perspective, as we cannot accurately incorporate patient-incurred costs (e.g., given variation in opportunity cost); however, we will track the frequency and duration of time spent by patients in intervention sessions and incorporate transportation time and costs. We will use a micro-costing approach recommended by the US Panel on Cost-Effectiveness in Health and Medicine, tracking all costs associated with implementing *Game Changers* as estimated from data collected from IDI Finance (e.g., facilitator compensation) and the team [43]. We will collect data using Drummond’s checklist of critical elements and follow best practices to maximize comparability of results between this and other studies [44]. The cost per resource will be calculated by multiplying the quantity used by unit cost; total cost will be derived by summating individual costs [45]. Fixed (capital) costs (e.g., overhead) will be allocated as the hours per week that premises are occupied for the intervention sessions. Capital costs will be annualized using a discount rate of 3% with an assumed lifespan of 40 years for physical infrastructure (e.g., buildings) and 10 years for equipment (e.g., laptops) [45]. The analysis will take into account all intervention session costs, but exclude costs incurred by staff and participants associated with the research components of the clinical trial.

Facilitators will record time spent on sessions using standardized templates, identifying each activity (e.g., training, preparation, sessions) and session-related materials (e.g., consumables such as paper). We will differentiate between intervention development (e.g., training) and recurrent costs of the intervention itself. Recurrent costs will be tracked to identify any cost efficiencies that are accrued over time. Within those, we will differentiate between fixed intervention costs and the marginal costs of adding new patients—in order to provide information on generalizability.

### Ethics and dissemination

The study protocol has been approved by the Institutional Review Board at The Infectious Diseases Institute (IDI) and the Human Subjects Protection Committee at RAND, as well as cleared by the Uganda National Council of Science and Technology as per national research regulations. Any protocol modifications will be submitted to the IRBs for review, and participants will be informed if warranted.

To ensure and maintain the scientific integrity of this human subject research project, and to protect the safety of its research participants, we have a three member Data Safety Monitoring Board (DSMB) that will intermittently (at 6-month intervals) monitor study adverse event data. The DSMB will be provided with periodic reports which include subject enrollment, subject retention, reasons for dropping out, and a listing of all adverse events that are plausibly related to the intervention or study procedures. Adverse events that are considered directly related to the intervention or other aspect of study participation will be reported immediately to the DSMB, the IRBs, and NIH. After review of the periodic reports, the DSMB will make a recommendation regarding the continuation, modification, or termination of the study to the study senior investigators, who will make the final decision regarding continuation or termination. All communications from the DSMB will be shared with the IRBs and NIH. To protect confidentiality, all research data will be kept in locked file cabinets and/or secure password protected computers and will be available only to members of the study team. Data will be identifiable only by study ID numbers. Personal information including participants’ name, address, and phone number will be stored separately from all research data. All data collected will be kept confidential and not shared with the client’s physician or other clinic staff or any of their social network members whom they may recruit to participate.

As a first step for dissemination, reporting results will be documented on ClinicalTrials.gov in accordance with NIH requirements on dissemination of clinical trial results. Information submitted will occur no later than 12 months after the primary completion date. The results produced by this investigation will be presented at international conferences and published in a timely fashion, ideally in the last year of the study period. All members of the study team will be eligible for authorship if they meet standard guidelines for contribution to the manuscript. All final peer-reviewed manuscripts that arise from this proposal will be submitted to the digital archive PubMed Central for open access. De-identified data, assessment and intervention materials, and analytic code will be made available upon request from external researchers and following review and approval of the study team.

## Discussion

This study will conduct a randomized, controlled evaluation of the social network-based group intervention, Game Changers, designed to empower PLWH to advocate for HIV protective behaviors among people in their social networks. This network approach to HIV prevention is particularly timely in the era of biomedical interventions, which require widespread penetration of effective HIV prevention and care messaging into communities. With the exception of our pilot work, we are unaware of studies of the effects of prevention advocacy by PLWH with family, friends, and community members. If successful, the intervention will be a significant innovation for the field, as it targets advocacy to all types of social network members rather than specific peer or risk groups. Furthermore, this is one of the few network-driven interventions to use social network data to inform intervention strategies for targeting advocacy to bridging and popular network members, which may optimize the knowledge and support transfer for HIV protective behaviors throughout a network.

Positioning PLWH as central to the solution for controlling (vs. causing) the HIV epidemic has the potential to reduce HIV stigma and improve prevention outcomes at the individual, household, and network levels. With essentially every family affected by HIV in high-prevalence settings like Uganda, the intervention can dramatically impact the fight against HIV and HIV prevention through widely disseminated and targeted advocacy. If successful, this intervention model has the potential to not only impact HIV, but also establish a paradigm that can be applied to other health conditions.

## Trial status

Protocol version 1.0 (January 10, 2022); recruitment began January 24, 2022, and is expected to be completed July 2023.

## Data Availability

De-identified dataset and statistical code are available to researchers upon submission of proposal and review by the study team.

## Abbreviations

PLWH: People living with HIV
HIV: Human immunodeficiency virus
ART: Antiretroviral therapy
USD: United States dollar
IDI: The Infectious Diseases Institute
NAFOPHANU: National Forum of People Living with HIV/AIDS Networks in Uganda
CAB: Community advisory board
AIDS: Acquired immunodeficiency syndrome
PrEP: Pre-exposure prophylaxis
ICC: Intra-cluster correlation
CEA: Cost-effectiveness analysis
IRB: Institutional review board
DSMB: Data safety monitoring board
NIH: National Institutes of Health
